# Matrix Metalloproteinase-8 Mediates the Unfavorable Systemic Impact of Local Irradiation on Pharmacokinetics of Anti-Cancer Drug 5-Fluorouracil

**DOI:** 10.1371/journal.pone.0021000

**Published:** 2011-06-09

**Authors:** Chen-Hsi Hsieh, Chia-Yuan Liu, Yen-Ju Hsieh, Hung-Chi Tai, Li-Ying Wang, Tung-Hu Tsai, Yu-Jen Chen

**Affiliations:** 1 Institute of Traditional Medicine, School of Medicine, National Yang-Ming University, Taipei, Taiwan; 2 Department of Radiation Oncology, Far Eastern Memorial Hospital, Taipei, Taiwan; 3 Department of Radiation Oncology, Mackay Memorial Hospital, Taipei, Taiwan; 4 Department of Gastrointestinal Division Mackay Memorial Hospital, Taipei, Taiwan; 5 Department of Medical Research, Mackay Memorial Hospital, Taipei, Taiwan; 6 Department of Education and Research, Taipei City Hospital, Taipei, Taiwan; 7 School and Graduate Institute of Physical Therapy, College of Medicine, National Taiwan University, Taipei, Taiwan; Technische Universität München, Germany

## Abstract

Concurrent chemoradiation with 5-fluorouracil (5-FU) is widely accepted for cancer treatment. However, the interactions between radiation and 5-FU remain unclear. Here, we evaluated the influence of local irradiation on the pharmacokinetics of 5-FU in rats. The single-fraction radiation was delivered to the whole pelvic fields of Sprague-Dawley rats after computerized tomography-based planning. 5-FU at 100 mg/kg was prescribed 24 hours after radiation. A high-performance liquid chromatography system was used to measure 5-FU in the blood. Matrix metalloproteinase-8 (MMP-8) inhibitor I was administered to examine whether or not RT modulation of 5-FU pharmacokinetic parameters could be blocked. Compared with sham-irradiated controls, whole pelvic irradiation reduced the area under the concentration versus time curve (AUC) of 5-FU in plasma and, in contrast, increased in bile with a radiation dose-dependent manner. Based on protein array analysis, the amount of plasma MMP-8 was increased by whole pelvic irradiation (2.8-fold by 0.5 Gy and 5.3-fold by 2 Gy) in comparison with controls. Pretreatment with MMP-8 inhibitor reversed the effect of irradiation on AUC of 5-FU in plasma. Our findings first indicate that local irradiation modulate the systemic pharmacokinetics of 5-FU through stimulating the release of MMP-8. The pharmacokinetics of 5-FU during concurrent chemoradiaiton therapy should be rechecked and the optimal 5-FU dose should be reevaluated, and adjusted if necessary, during CCRT.

## Introduction

Radiation therapy (RT) is used as an effective local treatment modality to inhibit cell proliferation, induce cell death and suppress tumor growth [1]. To improve the treatment outcome, in terms of both locoregional control and survival, the concurrent use of chemotherapy during radiation therapy (CCRT) is now the standard treatment for various malignancies, especially locally advanced cancers. Among the drugs used to enhance RT effect, 5-fluorouracil (5-FU) is one of the most commonly used chemotherapeutic agents of CCRT [2], [3], [4], [5].

In the past, RT was solely used as a local treatment and its effect was estimated by local effect model [6]. However, growing evidence shows that irradiation has direct DNA damage-dependent effects as well as sending signals to neighboring cells. The reactions of non-irradiated cells responding to signals produced by neighboring irradiated cells are termed the bystander effect [7], [8]. Furthermore, longer-range effects occurring within or between tissues are also reported and are termed abscopal, out-of-field or distant bystander responses [9]. Several molecules play roles in bystander signaling that involve stress responses and cell-cell signaling, however, none of them is specific to radiation exposure. Several studies show the alterations of plasma substance levels responding to radiation, such as interleukin 6 (IL-6) [10], IL-8 [11], transforming growth factor-beta 1 (TGF-β1) [12], tumor necrosis factor α (TNF-α) [13], reactive oxygen species [14] and reactive nitrogen species [15]. Yet, no strong evidence for causal relationships of these molecules is provided. Recently, we reported that abdominal irradiation could significantly modulate the systemic pharmacokinetics of 5-FU at 0.5 Gy, off-target area in clinical practice, and at 2 Gy, the daily treatment dose for target treatment in an experimental rat model [16]. Additionally, the results from a clinical investigation showed that colorectal cancer patients with lower AUC of 5-FU during adjuvant chemotherapy had lower disease-free survival [17]. Taken together, these lines of evidence support the importance and necessity to search for the mediators responsible for the unexpected effect of local RT on systemic pharmacokinetics of chemotherapeutic agents, such as 5-FU. In the present study, we investigated possible soluble mediators involved in the effect of localized whole pelvic RT, with liver sparing, on the pharmacokinetics of 5-FU in rats.

## Results

### Plasma pharmacokinetic parameters of 5-FU and whole pelvic irradiation

**Figure 1:**
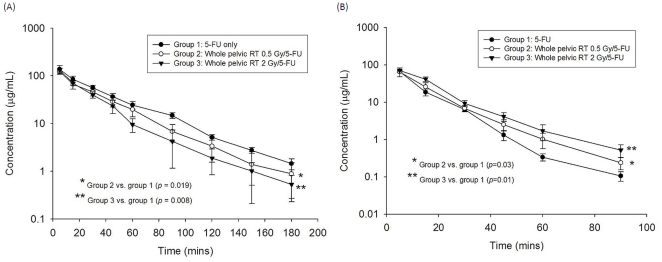
The area under the concentration vs. time curve (AUC) of 5-fluorouracil (5-FU) 100 mg/kg administered to rats in the control, 0.5-Gy and 2-Gy groups. The transverse axis illustrates time in minutes and the vertical axis represents the concentration of 5-FU. (A) Plasma. (B) Bile. Each group's data was collected from six rats.

To verify that local RT modulated the systemic pharmacokinetics of 5-FU, we established an experimental model using CT-based planning and pelvic irradiation in rats, and integrated it into a pharmacokinetic assay system. Intriguingly, we found that pelvic irradiation markedly reduced the AUC of 5-FU in rats by 17.6% at 0.5 Gy (*P* = 0.019) and 21.5% at 2 Gy (*P* = 0.008) (Fig. 1A). Of special interest, the radiation at 2 Gy to the rat whole pelvis simulated the daily treatment dose to a human, whereas the low-dose radiation (0.5 Gy) simulated the dose deposited in the generous, off-target area in clinical practice. As shown in Table 1, pelvic irradiation significantly decreased mean residence time (MRT), and by contrast, increased the clearance value of 5-FU when compared to non-irradiated controls. There was no significant difference in the values of half-life (T_1/2_), maximum observed plasma concentration (Cmax) or volume of distribution at steady state (Vss) within the tested groups.

**Table 1:** Plasma pharmacokinetics of 5-fluorouracil (100 mg/kg, i.v.) in rats after whole pelvic irradiation with or without 0.5 Gy or 2 Gy.

Parameters	Controls	Whole pelvic irradiation	
	0 Gy	0.5 Gy	2 Gy
AUC (min µg/mL)	4725±402	3893±329*	3711±484*
t_1/2_ (min)	34.7±9.6	24.7±3.5	29.9±7.1
Cmax (µg/mL)	164±36	167±23	179±19
MRT (min)	36.5±3.0	30.9±3.7*	25.0±4.8*
CL (mL/kg/min)	21.3±1.9	25.8±2.0*	27.3±3.6*
Vss (mL/kg)	778±88	775±116	670±60

AUC: area under the plasma concentration *vs.* time curve; t_1/2_: terminal elimination phase half-life; Cmax: maximum observed plasma concentration; MRT: mean residence time; CL: total plasma clearance; Vss: volume of distribution at steady state.

*The mean difference is significant at the 0.05 level in comparison to the control group.

### Bile pharmacokinetic parameters of 5-FU and whole pelvic irradiation

We found that pelvic irradiation markedly increased the AUC of 5-FU in bile of rats by 25.1% at 0.5 Gy and 30.6% at 2 Gy (Fig. 1B). Pelvic irradiation significantly decreased Cmax and clearance value, and in contrast, increased MRT and Vss of 5-FU, when compared to non-irradiated controls. Of interest, 2-Gy irradiation decreased Cmax, and in contrast, increased MRT (*P* = 0.008) and Vss (*P* = 0.015) of 5-FU to an extent greater than that of the 0.5-Gy group. There was no statistically significant difference between the 0.5-Gy and control groups for Cmax and Vss. Furthermore, no significant difference in T_1/2_ was noted among the three groups (Table 2).

**Table 2:** The bile of 5-Fluorouracil (100 mg/kg, i.v.) pharmacokinetics in rats after whole pelvic irradiation with and without 0.5 and 2 Gy.

Parameters	Controls	Whole pelvic irradiation	
	0 Gy	0.5 Gy	2 Gy
AUC (min µg/mL)	1180±49	1477±78*	1540±101*
t_1/2_ (min)	9.2±0.4	10.3±0.5	12.9±2.8
Cmax (µg/mL)	129±22	120±14	93±6.0* †
MRT (min)	8.9±1.1	11.1±0.5*	13.9±1.3* †
CL (mL/kg/min)	84.8±3.6	67.9±3.6*	65.1±4.3*
Vss (mL/kg)	754±102	753±25	903±55*

AUC: area under the plasma concentration *vs.* time curve; t_1/2_: terminal elimination phase half-life; Cmax: maximum observed plasma concentration; MRT: mean residence time; CL: total plasma clearance; Vss: volume of distribution at steady state.

*The mean difference is significant at the 0.05 level in comparison to the control group.

† The mean difference is significant at the 0.05 level between the 0.5 and 2 Gy groups.

### Hepatic function after RT or 5-FU treatment

**Figure 2:**
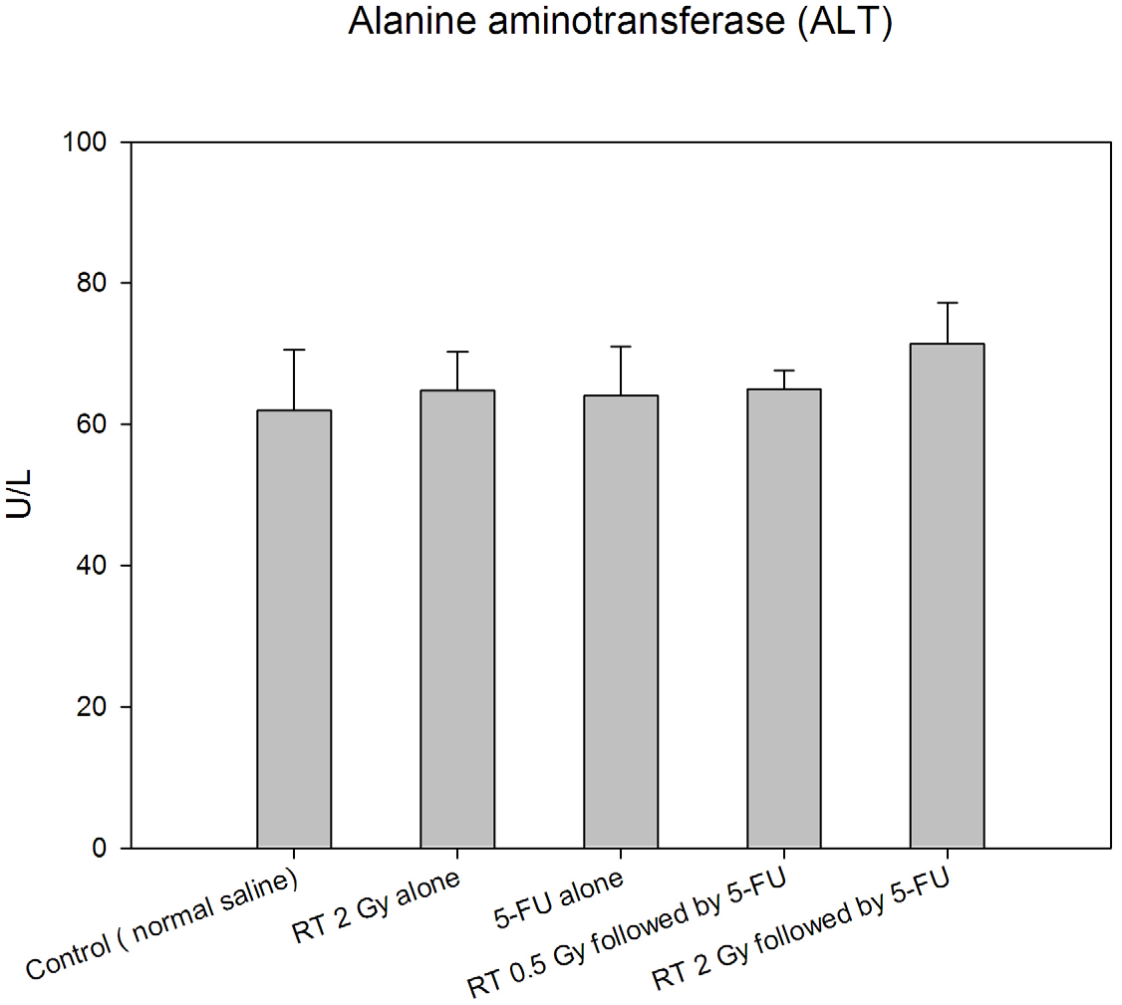
Plasma alanine aminotransferase (ALT) levels in rats of the control, 5-FU-treated only, 2Gy-treated only, 0.5 Gy followed by 5-FU-treated and 2 Gy followed by 5-FU-treated groups. The serum concentrations of alanine aminotransferase (ALT) levels were not significantly different between all tested groups. N = 5 for each group.

The serum concentrations of alanine aminotransferase (ALT) levels were no significant difference between the 5-FU-treated, 2Gy-treated, 0.5 Gy followed by 5-FU-treated and 2 Gy followed by 5-FU-treated and control groups (Fig. 2).

### The cytokines respond to RT or 5-FU in the plasma

**Figure 3:**
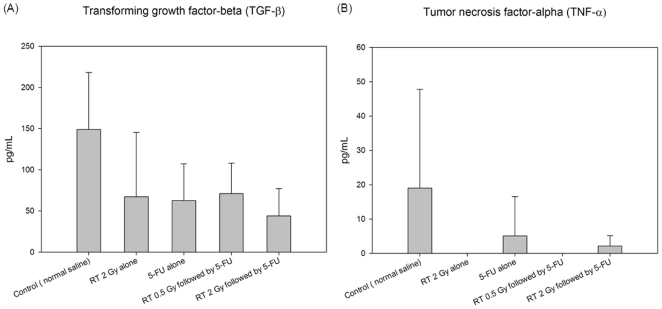
The cytokines respond to irradiation (RT) and 5-fluorouracil (5-FU) in plasma for control, RT 2 Gy alone, 5-FU alone, RT 0.5 Gy followed by 5-FU and RT 2 Gy followed by 5-FU groups. (A) The level of transforming growth factor beta 1 (TGF-β1) in plasma. (B) The level of tumor necrosis factor alpha (TNF-α) in plasma. Each group's data was collected from five rats.

Compared with the control group, there were no significant differences between the RT 2Gy alone, 5-FU alone, RT 0.5 Gy followed by 5-FU and RT 2 Gy followed by 5-FU group in the levels of transforming growth factor-beta 1 (TGF-β1) and tumor necrosis factor α (TNF-α) (Fig. 3A and 3B).

### Alteration of soluble factors in plasma caused by whole pelvic irradiation

**Figure 4:**
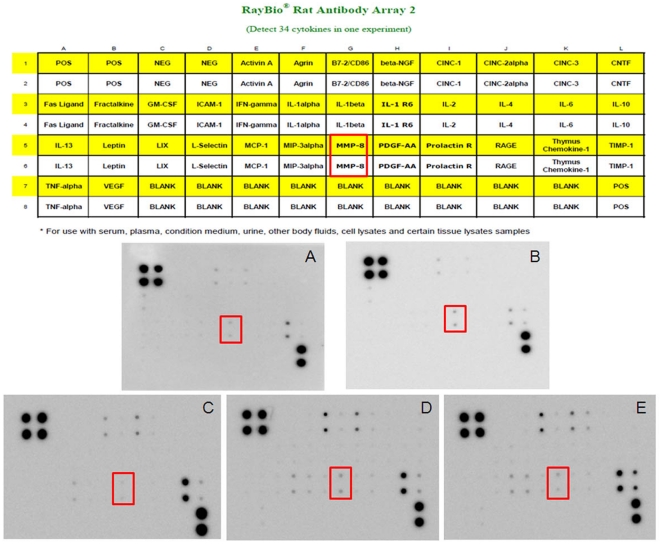
Cytokine profile of rat plasma treated with whole pelvic irradiation. The amount of matrix metalloproteinase-8 (MMP-8) increased in 0.5-Gy and 2-Gy irradiated groups when compared with the control group. A map of the locations of cytokine antibodies spotted onto the protein chip is shown on the up side of the Figure. Dotted squares indicate the location of MMP-8. Each cytokine is represented by duplicate spots in the locations shown. Cytokine antibody arrays assay of (A) untreated control group, (B) whole pelvic irradiation (RT) with 2 Gy only, (C) 5-fluorouracil (5-FU) alone, (D) RT with 0.5 Gy followed by 5-FU and (E) RT with 2 Gy followed by 5-FU. The cytokine array image represents results of one of three independent experiments, which show similar patterns of expression.

**Figure 5:**
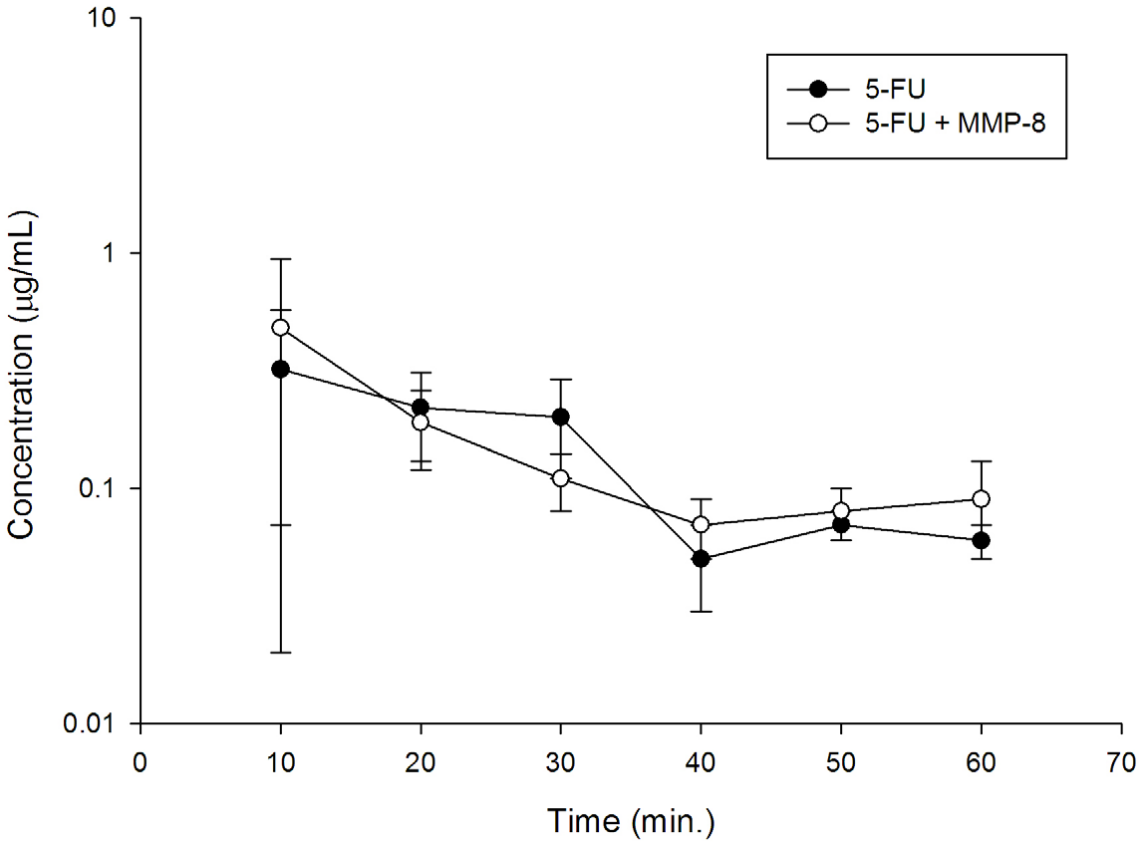
Intracellular 5-fluorouracil (5-FU) levels for 50 µM 5-FU treated HepG2 (Human liver tumor-derived cells possessing biochemical profiles characteristic of normal hepatocytes) with or without 10 µg/mL recombinant MMP-8 by high performance liquid chromatography.

To assess the changes in profile of soluble factors involved with whole pelvic irradiation, rat plasma samples were collected and subjected to a cytokine antibody array assay (Fig. 4). In comparison with control (untreated) group (Fig. 4A) and 5-FU alone group (Fig. 4C), the observable dose-dependent changes in plasma levels of soluble factors included matrix metalloproteinase-8 (MMP-8), monocyte chemoattractant protein 1 (MCP-1), cytokine-induced neutrophil chemoattractant -1 (CINC-1) and tissue inhibitor of metalloproteinase-1 (TIMP-1). Among these factors, only the increase in MMP-8 level was consistent in triplicate experiments. Compared with control groups, RT 2Gy alone could increase the expression of MMP-8 by factors of 3.8 (Fig. 4B). When compared with 5-FU alone group, the plasma levels of MMP-8 increased in both the RT 0.5 Gy followed by 5-FU (Fig. 4D) and RT 2 Gy followed by 5-FU groups (Fig. 4E) in a dose-dependent manner by factors of 2.8 and 5.3, respectively.

### Intracellular 5-FU levels with or without recombinant MMP-8 by high performance liquid chromatography

We examined the role of recombinant MMP-8 on intracellular concentration of 5-FU in HepG2 (Human liver tumor-derived cells possessing biochemical profiles characteristic of normal hepatocytes) without receiving radiation. There were no significant effect on the AUC of 5-FU between the recombinant MMP-8 plus 5-FU and 5-FU alone groups (Fig. 5). We found that recombinant MMP-8, not induced by irradiation, will not influence intracellular concentration of 5-FU in liver cells, mimicking a pharmacokinetic changes at cellular level.

### Modulation of 5-FU pharmacokinetic by irradiation was reversed by MMP-8 inhibitor

**Figure 6:**
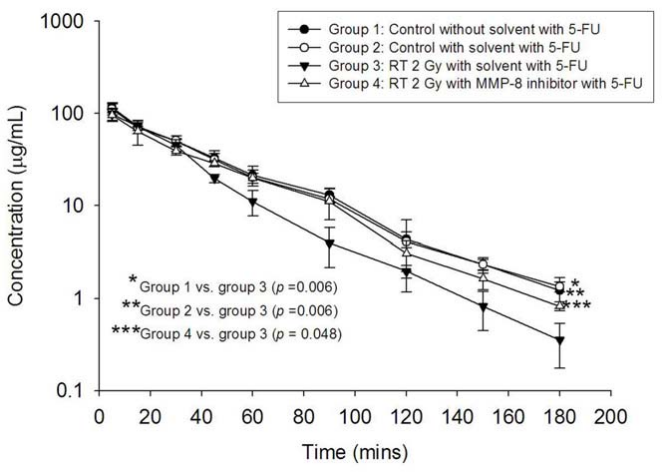
The plasma concentration versus time curve (AUC) of 5-FU post irradiation was reversed by the matrix metalloproteinase-8 (MMP-8) inhibitor. The area under the plasma concentration versus time curve (AUC) of 5-FU 100 mg/kg administered to rats in the control group without solvent, control group with solvent, whole pelvic 2-Gy irradiation with solvent, and whole pelvic 2-Gy irradiation with solvent and MMP-8 inhibitor. The transverse axis illustrates time in minutes and the vertical axis represents the concentration of 5-FU in the plasma. Each group's data was collected from four rats.

We next examined the role of MMP-8 on the effect of RT on 5-FU pharmacokinetics using an MMP-8 inhibitor. Neither the MMP-8 inhibitor alone nor its vehicle had a significant effect on the AUC of 5-FU in comparison with the controls (Fig. 6). We found that pretreatment with MMP-8 inhibitor significantly attenuated the decline in AUC of 5-FU caused by pelvic irradiation (AUC_irradiation_ versus AUC_MMP-8 inhibitor+irradiation_ was 3305 versus 3963 min µg/mL, *P*<0.05). Moreover, the decreased MRT and increased clearance value caused by irradiation were completely reversed by use of the MMP-8 inhibitor (Table 3).

**Table 3:** The plasma of 5-Fluorouracil (5-FU) (100 mg/kg, i.v.) pharmacokinetics in rats treated with and without MMP-8 inhibitor then delivered to whole pelvic irradiation with 2 Gy.

Parameters	Controls(withoutsolvent)	Controls (withsolvent)	Whole pelvicIrradiation 2 Gy	
			with solvent	with solvent and MMP-8 inhibitor
	5-FU			
AUC (min µg/mL)	4285±215*	4285±141*	3305±28	3963±427*
t_1/2_ (min)	33.3±16.1	27.8±3.0	31.0±8.8	32.3±9.9
Cmax (µg/mL)	151±17	146±35	132±51	122±29
MRT (min)	37±1.7*	36±3.7*	26±3.8	36±2.1*
CL (mL/kg/min)	23.4±1.1*	23.4±0.8*	30.3±0.3	25.4±2.8*
Vss (mL/kg)	859±22	836±97	784±110	911±127

AUC: area under the plasma concentration *vs.* time curve; t_1/2_: terminal elimination phase half-life; Cmax: maximum observed plasma concentration; MRT: mean residence time; CL: total plasma clearance; Vss: volume of distribution at steady state.

*The mean difference is significant at the 0.05 level in comparison to the whole pelvic irradiation with solvent and 5-FU group.

## Discussion

After proof of the concept that local RT affected systemic pharmacokinetics of chemotherapeutics using 5-FU as a model, we next screened for possible soluble factors responsible for this effect, which was identified as MMP-8. MMP-8, also known as collagenase-2 or neutrophil collagenase, is a member of the zinc-dependent interstitial collagenase subgroup of the MMP family of neutral proteinases [18]. We demonstrated that MMP-8 possessed unexpected bioactivity in modulating the pharmacokinetics of 5-FU.

Polymorphonuclear neutrophils (PMNs) are the main source of MMP-8 in humans and mice [19], [20]. MMP-8 is stored in the granules of PMNs and is released upon degranulation [21]. Fisher *et al*. [22] reported that MMP-8 protein levels in human skin were increased approximately 4-fold within 8 h and remained elevated for 24 h after ultraviolet irradiation. Radiation treatment at tumor bearing sites induces inflammation in the irradiated field and recruits T lymphocytes, neutrophils, lymphocytes, macrophages, plasma cells and dendritic cells [18], [23], [24]. Additionally, irradiation induces up-regulation of the genes of the main proinflammatory chemokines [25]. In the present study, we irradiated the whole pelvis of rats, which could have delivered a radiation dose to circulating neutrophils, tissue and bone marrow macrophages and within the pelvis. Collectively, this raises the possibility that pelvic irradiation could stimulate neutrophils and/or the other inflammatory cell ontogeny, induce inflammatory stress, and enhance the secretion of MMP-8 (Fig. 4) as well as various other proinflammatory mediators. Additionally, several studies show the alterations of plasma substance levels responding to radiation, such as TGF-β1 [12] and TNF-α [13]. Moreover, TGF-β could be a target for 5-FU via c-Jun NH2-terminal kinase/activator protein-1 activation in human fibroblasts [26]. In addition, TNF-α involves in the regulation of fluoropyrimidine-activating enzymes, Uridine phosphorylase (UPase), which induces UPase gene expression with consequent improvement of 5-FU antiproliferative activity [27]. However, in the current study, the levels of TGF-β1 and TNF-α are not increased in the RT alone, 5-FU alone or RT followed by 5-FU groups when compared with control group (Fig. 3A and 3B). These data suggest that MMP-8 appears to play a major role in local RT-induced modulation of systemic 5-FU pharmacokinetics but not through these cytokines.

Harty MW et al. [28] reported that polymorphonuclear cell-derived MMP8 plays an important role for liver repair in their reversible biliary obstruction model. The current study shows that the application of pelvic RT or 5-FU would not cause the damages of liver function. Thus, the response of MMP-8 induced by RT in RT-PK phenomena would not be the process of inflammatory infiltration to liver such as severe damage caused by biliary obstruction. Besides, there are no differences of viability (data not show) and intracellular 5-FU levels (Fig. 5) for 5-FU-treated HepG2 with or without recombinant MMP-8. HepG2 cells maintain many of the morphological and biochemical characteristics of normal hepatocytes, such as the secretion of most plasma proteins expected from liver cells, including apolipoprotein B [29]. The result suggests that recombinant MMP-8, not induced by irradiation, will not influence the PKs of 5-FU in liver cells and has no significant toxicity to human hepatoblastoma-derived cell line, HepG2. Taken together, MMP-8 may not modulate the liver function.

Another important issue is the similar, but lesser, effect of low-dose RT on 5-FU pharmacokinetics (Fig. 1). Body distribution of low-dose RT (0.5 Gy, for example) in clinical practice becomes greatly generous with advanced radiotherapy techniques and modalities, such as intensity-modulated radiotherapy, helical tomotherapy, rapid arc radiotherapy, volumetric modulated arc therapy and others, in comparison with conventional RT. While accepting the benefits of targeting the tumors and sparing the critical organs by using these advanced techniques [30], [31], that one remains cautious the generous, low-dose irradiation could produce unexpected or unwanted biological effects. Clinically, we previously observed that low-dose, off-target radiation delivered by highly conformal tomotherapy could cause severe toxicity to the critical organs around the targets, such as lungs, and cause radiation pneumonitis [32]. Yet, the medical community has no comprehensive understanding regarding the biological effects of generous, low-dose RT. We hope this study will increase our knowledge of these effects and provide an experimental model to understand the biological effects of generous, low-dose RT in the era of highly conformal RT.

**Figure 7:**
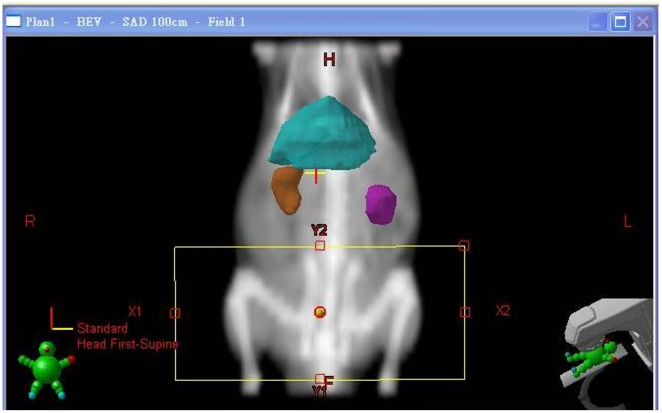
Computed tomography was used for simulation of the whole pelvic field. The cranial margin was set at the top of bilateral iliac crest for the whole pelvic field. Conventional radiotherapy was used to deliver the radiation dose via the anterior-posterior (AP) and PA portals.

About 80% of 5-FU is catabolyzed by the liver via the dihydropyrimidine dehydrogenase (DPD) pathway to generate toxic 5-fluoro-5,6-dihydro-uracil, whereas the anabolic pathway, via orotate phosphoribosyl transferase, produces active metabolites including 5-fluorouridine-5′-monophosphate, 5-fluorouridine, and 5-fluoro-2′-deoxyuridine [33], [34]. The overall toxicity was twice as high in patients with profound DPD deficiencies (<45% of the mean DPD activity of a control population) when compared to patients with moderate DPD deficiencies (between 45% and 70% of the mean DPD activity of a control population), as reported by Milano and the coauthors [35]. Because 10% to 20% of 5-FU is excreted unchanged in the urine [36], for patients with renal dysfunction, the plasma concentration of 5-FU on nondialysis days is significantly higher than on dialysis days, and this may be due to the removal of some factors from plasma by hemodialysis, which inhibit DPD activity [37]. In addition, 5-FU has a relatively narrow therapeutic index, a strong correlation is described between exposure to 5-FU and both hematologic and gastrointestinal toxicity [38]. Thus, if the liver or kidneys fall into the irradiated volume,[16] DPD, a rate limiting step in the catabolism of 5-FU [39], may be affected by radiation injury to liver or kidneys. However, in the current study, the liver and kidneys are excluded from the whole pelvic irradiation field (Fig. 7). In addition, the ALT levels were not significantly different between the control, 5-FU-treated, or pelvic RT groups with or without 5-FU. (Fig. 2) Thus, the effect of RT on AUC of 5-FU noted in this study may not be caused by direct modulation of liver function by RT or 5-FU.

Compared with the control group, whole pelvic irradiation decreased the AUC of 5-FU in the plasma to a statistically significant level (Fig. 1A). In contrast, irradiation increased the AUC of 5-FU in the bile significantly (Fig. 1B). It was accompanied by a reduction in MRT and increase in clearance value in the plasma, but an increase in MRT and reduction of clearance value in the bile. With respect to pharmacokinetics, this suggests that pelvic irradiation could facilitate the excretion of 5-FU.

Given that the concurrent use of chemotherapeutics in combination with localized conformal RT improves clinical treatment outcomes for an increasing number of malignancies [2], [3], [4], [5], our results show that both localized target-in and generous target-off irradiation could affect 5-FU pharmacokinetics, and provides a reason for considering the adjustment of chemotherapeutic administration during the RT course. The effect of localized RT on systemic pharmacokinetics of chemotherapeutic agents or the other drugs clearly needs further clinical evaluation.

## Materials and Methods

### Materials and reagents

The 5-FU and high-performance liquid chromatography (HPLC)-grade methanol were purchased from Sigma Chemicals (St. Louis, MO, USA) and Tedia Company, Inc. (Fairfield, OH, USA), respectively. Milli-Q grade water (Millipore, Bedford, MA, USA) was used for the preparation of solutions and mobile phases.

### Animals and sample preparation

Adult, male Sprague-Dawley rats (300±20 g body weight) were provided by the Laboratory Animal Center at National Yang-Ming University (Taipei, Taiwan). They were housed in a specific pathogen-free environment and had free access to food (Laboratory Rodent Diet 5001, PMI Nutrition International, LLC, MO, USA) and water. All experimental animal surgery procedures were reviewed and approved by the animal ethics committee of Mackay Memorial Hospital, Taipei, Taiwan (MMH-A-S-98011).

The rats were anesthetized with urethane 1 g/ml and α-chloralose 0.1 g/ml (1 ml/kg by intraperitoneal injection), and were immobilized on a board when undergoing computed tomography for simulation of the whole pelvic field. The cranial margin was set at the top of the bilateral iliac crests for the whole pelvic field (Fig. 7). Conventional radiotherapy was used to deliver the radiation dose via the anterior-posterior (AP) and PA portals. The experimental animals were randomized to the control, 2-Gy alone, 5-FU alone, 0.5-Gy followed by 5-FU, and 2-Gy followed by 5-FU groups. Each group's data was collected from six rats.

Allometric scaling of the radiation doses (0.5 and 2 Gy) between humans and rats, respectively, was an important consideration in the study. The reason for the use of 0.5 and 2 Gy for the rats was to simulate the relevant dosage range for daily treatment of the human torso, for safety and workability, as previously reported [16]. Briefly, there was no direct comparison of allometric scaling using whole pelvic irradiation. Nonetheless, the allometric scaling of the lethal dose (LD50) (Gy) of total-body irradiation for humans and rats is 4 Gy and 6.75 Gy, respectively [40]. Given that this difference is moderate, we decided to use 0.5 and 2 Gy for rats to simulate the relevant dose range for daily treatment of the human torso.

Ambre *et al*. [41] studied the elimination of 5-FU and its metabolites after intravenous administration of 5-FU at 15 and 150 mg/kg to rats. The results of that study suggested that saturation of the catabolic pathway occurred after the higher dose. Jarugula *et al*. [42] proved that the dose-normalized area under the curve (AUC) was significantly higher after administration of 100 mg/kg (mean ± standard deviation, SD, 1.14±0.55 mg·h/L/mg) than after 50 mg/kg (mean ± SD, 0.50±0.16 mg·h/L/mg) or 10 mg/kg (mean ± SD, 0.43±0.11 mg·h/L/mg). Thus, we chose 100 mg/kg as a feasible 5-FU dose in rats for examination of 5-FU pharmacokinetic parameters, based on previous reports [16], [41], [42].

Twenty hours after RT, the rats were administered 100 mg/kg of 5-FU in 2 mL of normal saline by intravenous infusion into the femoral vein over a 2-min period [42]. A 150-µL blood sample was withdrawn from the jugular vein with a fraction collector according to a programmed schedule at 5, 15, 30, 45, and 60 min, and 1.5, 2, 2.5, and 3 h following drug administration. The blood samples were immediately centrifuged at 3300×*g* for 10 min. The resulting plasma (50 µL) was added to 1 mL of ethyl acetate in a clean tube, vortexed for 5 min, and centrifuged at 5900×*g* for 10 min. After centrifugation, the upper organic layer containing the ethyl acetate was transferred to a new tube and evaporated to dryness under flowing nitrogen. The dried residue was reconstituted with 50 µL of Milli-Q water (Millipore). A 20-µL aliquot of the solution was injected to the high performance liquid chromatography-ultraviolet (HPLC-UV) detection system.

### High performance liquid chromatography

Chromatographic analysis was performed on a Model LC-20AT HPLC system (Shimadzu, Tokyo, Japan) equipped with a Model SPD-20A wavelength UV detector, SIL-20AC autosampler, and an LC Solution data processing system. A LiChroCART RP-18e column (Purospher, 250 mm, 5 µm, Merck, Darmstadt, Germany) with a LiChroCART 4-4 guard column was used for separation. The mobile phase comprised 10 µM potassium phosphate-methanol (99∶1, v/v, pH 4.5 adjusted by 85% phosphoric acid), and the flow rate of the mobile phase was 1 ml/min. The detection wavelength was set at 266 nm. Under these conditions, the retention time of 5-FU was 5.4 min. The linearity of calibration curves was demonstrated by the good determination coefficients (*r* ^2^) obtained for the regression line. Good linearity was achieved over the range of 0.01–5 µg/ml, with all coefficients of correlation greater than 0.998. All samples were freshly prepared, including the standard solutions, from the same stock solution (5 mg/mL). The 0.01-µg/mL limit of quantification was defined the lowest concentration on the calibration curve that could be measured routinely with acceptable bias and relative SD. The overall mean precision, defined by the relative SD, ranged from 0.2% to 11.0%. Analytical accuracy was expressed as the percentage difference of the mean observed values compared to known concentrations varying from −10.0% to 14.0%. The recovery results for concentrations of 0.1–10 µg/mL were 92.0%–94.0%

### Evaluation of hepatic functions

The plasma levels of alanine aminotransferase (ALT) were measured to check the influence of different modalities for hepatic function by a standard colorimetric method using a Synchron LX20 spectrophotometer (Beckman Coulter) and manufacturer-supplied reagents.

### Serum cytokine analysis

The plasma levels of cytokines (transforming growth factor beta 1 (TGF-β1) and tumor necrosis factor alpha (TNF-α)) obtained from the mouse blood samples were analyzed using enzyme-linked immunosorbent assay (ELISA) (R&D Systems) according to the manufacturer's instructions.

### Cytokine antibody array

The rat plasma samples were analyzed using the a cytokine antibody array (RayBio**®** Mouse Cytokine Antibody Arrays II, RayBiotech, Inc., Norcross, Ga.) according to the manufacturer's instructions and as previously described [43] to detect possible mediators of the 5-FU–RT interaction. This particular array simultaneously detects 34 murine cytokines. (Fig. 4) Briefly, cytokine array membranes were blocked in 2 ml of 1× blocking buffer for 30 min and then incubated with 1 ml of plasma sample at room temperature for 1–2 h. Samples were then decanted from each container, and the membranes were washed three times with 2 ml of l× wash buffer I, followed by two washes with 2 ml of l× wash buffer II at room temperature with shaking. Membranes were then incubated in 1∶250 diluted biotin-conjugated primary antibodies at room temperature for 1–2 h and washed as described above, before incubation in 1∶1000 diluted horseradish peroxidase (HRP)-conjugated streptavidin. After incubation in HRP-conjugated streptavidin for 30–60 min, membranes were washed thoroughly and exposed to a peroxide substrate (detection buffers C and D, RayBiotech, Inc.) for 5 min in the dark before imaging. Membranes then were exposed to X-ray film (Kodak X-OMAT AR film) at room temperature for 1 minute. Signal intensities were analyzed with Fuji Film Multi Gauge V2.1. Biotin-conjugated IgG served as a positive control at six spots, where it was used to identify membrane orientation and to normalize the results from different membranes that were being compared. For each spot, the net optical density was determined by subtracting the background optical density from the total raw optical density and the optical density of each cytokine was represented as a percentage of the positive control.

### Determination of intracellular 5-FU levels with or without recombinant MMP-8 by high performance liquid chromatography

To examine the effect of MMP-8 on normal liver cells, a human hepatoblastoma-derived cell line, HepG2, was used to simulate normal liver cells in our additional experiments. After treatment with 10 µg/mL recombinant MMP-8 plus 50 µM 5-FU or 50 µM 5-FU alone, HepG2 (Human hepatocellular carcinoma cell line) cells were collected every 10 min until 60 min and washed twice in phosphate-buffered saline (PBS) followed by centrifugation at 13000 rpm for 5 min. To extract intracellular 5-FU, an extraction solution containing 0.1% Triton X-100 in dimethyl sulfoxide was added to the cell pellets, and this suspension was vortexed and centrifuged at13000 rpm for 5 min. The resulting solution was mixed with an equal volume of acetonitrile for deproteination. The protein precipitates were removed by centrifugation (13000 rpm for 5 min), and a 20 µL aliquot of the supernatant was subjected to high performance liquid chromatography analysis as mention before.

### Mediator inhibitor preparation and experiment

MMP-8 inhibitor I (Calbiochem, La Jolla, CA) was administered to the rats to examine whether or not RT modulation of 5-FU pharmacokinetic parameters could be blocked. Briefly, MMP-8 inhibitor I was dissolved in PEG400/ethanol [4∶1 (v/v)] solution, yielding a final concentrations of 5 mg/mL. Two hours before irradiation, 10 mg/kg of MMP-8 inhibitor I was infused into the rat's tail vein over a 2-min period. After that, the rats were anesthetized with urethane 1 g/ml and α-chloralose 0.1 g/ml (1 ml/ kg by intraperitoneal injection), and were immobilized on a board to undergo computed tomography for simulation of the whole pelvic field and received irradiation, as described previously. The experimental animals were randomized to control without PEG400/ethanol [4∶1 (v/v)] solution (0 Gy), control with solvent (0 Gy), whole pelvic irradiation (2 Gy) with solvent and whole pelvic irradiation (2 Gy) with MMP-8 inhibitor and solvent groups, respectively. After RT sham RT 20 hrs, all rats received 5-FU (100 mg/kg) injections and the pharmacokinetic parameters of 5-FU were analyzed. Each group's data was collected from four rats.

### Pharmacokinetics and data analysis

Pharmacokinetic parameters including the AUC for concentration versus time, terminal elimination phase t_1/2_, Cmax, MRT, total plasma clearance and Vss were calculated using the pharmacokinetics calculation software WinNonlin Standard Edition, Version 1.1 (Scientific Consulting, Apex, NC, USA) using a compartmental method.

### Statistical methods

The results are presented as means ± standard deviations. Differences in actuarial outcomes between the groups were calculated using one-way analysis of variance (ANOVA), with post hoc multiple comparisons. All analyses were performed using the SPSS, version 12.0 (SPSS, Chicago, IL, USA).
